# Jörg Langowski: his scientific legacy and the future it promises

**DOI:** 10.1186/s13628-018-0045-1

**Published:** 2018-07-16

**Authors:** Giuseppe Chirico, Alexander Gansen, Sanford H. Leuba, Ada L. Olins, Donald E. Olins, Jeremy C. Smith, Katalin Tóth

**Affiliations:** 1 Dipartimento di Fisica, Università di Milano-Bicocca, Milan, Italy; 2 Biophysics of Macromolecules (B040), Deutsches Krebsforschungszentrum, Im Neuenheimer Feld 280, 69120 Heidelberg, Germany; 3 Departments of Cell Biology and Bioengineering, 2.26a UPMC Hillman Cancer Center, University of Pittsburgh School of Medicine, 5117 Centre Avenue, Pittsburgh, PA 15213 USA; 4 Department of Pharmaceutical Sciences, College of Pharmacy, University of New England, Portland, ME USA; 5 Oak Ridge National Laboratory, P.O. Box 2008 MS6309, Oak Ridge, TN 37831-6309 USA; 6 Department of Biochemistry and Cellular and Molecular Biology, University of Tennessee, M407 Walters Life Sciences, 1414 Cumberland Avenue, Knoxville, TN 37996 USA

## Abstract

**Background:**

With the passing of Jörg Langowski 6 May 2017 in a sailplane accident, the scientific community was deprived of a strident and effective voice for DNA and chromatin molecular and computational biophysics, for open access publishing and for the creation of effective scientific research networks.

**Methods:**

Here, after reviewing some of Jörg’s key research contributions and ideas, we offer through the personal remembrance of his closest collaborators, a deep analysis of the major results of his research and the future directions they have engendered.

**Conclusions:**

The legacy of Jörg Langowski has been to propel a way of viewing biological function that considers living systems as dynamic and in three dimensions. This physical view of biology that he pioneered is now, finally, becoming established also because of his great effort.

## Background

**Fig. 1:**
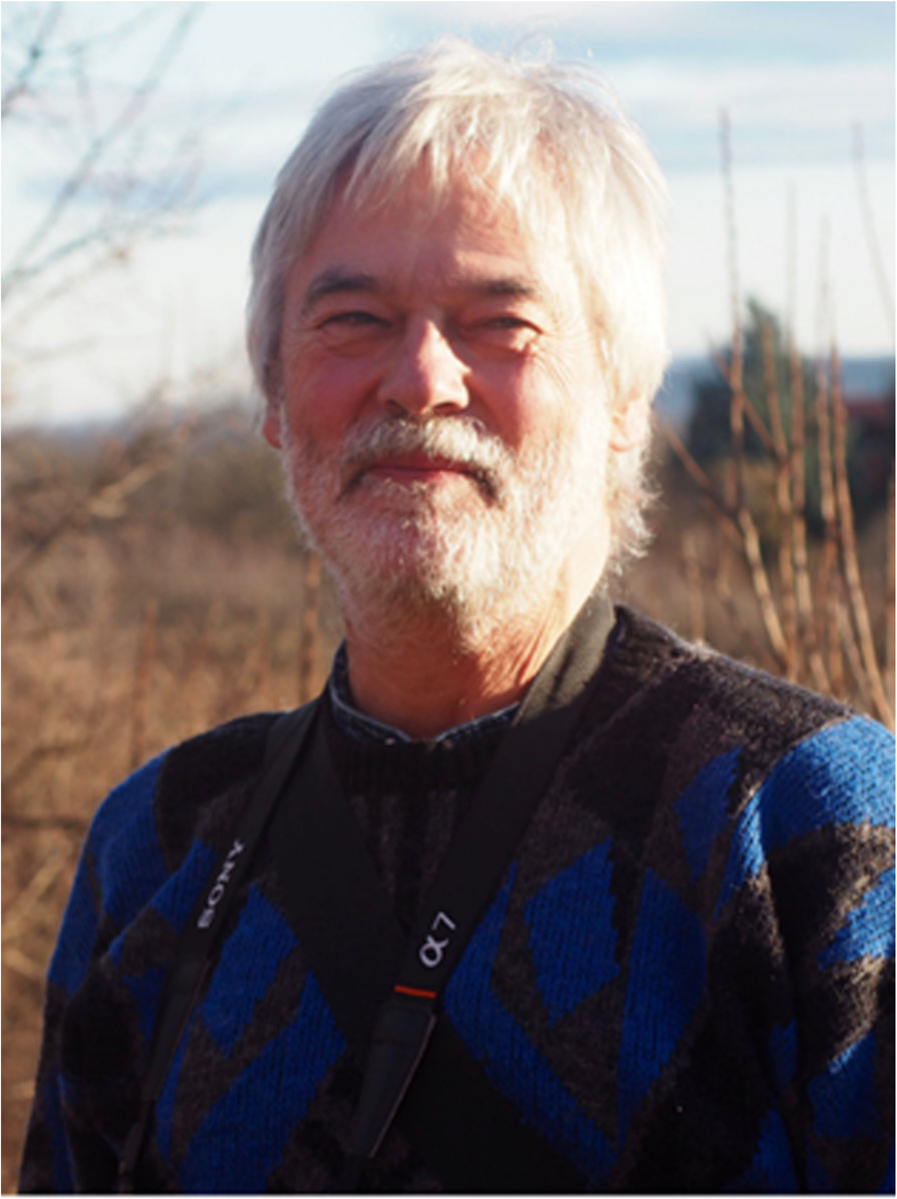
Jörg, Langowski. Jörg, December 2016 in Hungary. Photograph taken by Eva Langowski

Jörg (Fig. 1) studied biochemistry in Hannover, which was just emerging as a science hub. A versatile student, he also studied physics, electrical engineering and computer science in depth. His biochemical knowledge provided later solid impetus to his biophysical experiments and simulations. He wrote his diploma thesis on the study of nucleic acid melting, for which he complemented his experiments with simulations programmed on the computers of that time. He completed his doctoral thesis, also in Hannover, under the direction of Prof. Günter Maass and Claus Urbanke. In the doctoral thesis, defended in 1977, Jörg constructed a pulsed quench-flow apparatus with a machine code programmable microprocessor to study the biophysics of enzyme-DNA recognition.

Between these two theses his adventurous spirit carried him to Stanford, where for one year he worked in the laboratory of Professor Buzz Baldwin. This stay resulted in his most cited paper, about mini DNA circles [1]. After his Ph.D., he returned to the USA, this time to Seattle, where he made everlasting friends not only with Mickey Schurr, his boss, but also with light scattering, a method by which the fluctuations of randomly moving molecules are followed in free solution providing information about their structure. A perfect match for an adventurer. It was in Mickey’s lab that Jörg started working on superhelical DNA. It was also in Seattle that he committed to the Macintosh computer forever; he was a founding member of and for 10 years an author at the software developing journal Mac-tutor.

His first opportunity to assemble a research group occurred at the EMBL Outstation in Grenoble, in 1985. Inheriting a light scattering setup, originally used to test protein aggregations for the crystallization, in the following 9 years Jörg studied the structure of superhelical, curved and other interesting DNA structures with a small, but growing group of doctoral students and postdocs, including Werner Kremer, Giberto Chirico (see his contribution later in the text) and Kostya Klenin. Additionally, the group developed simulation methods to better approach the nature of flexible polymers, and Jörg evolved a maximum entropy procedure for data treatment in scattering methods. Questions about DNA superhelicity are still not exhausted, even after 30 years.

**Fig. 2:**
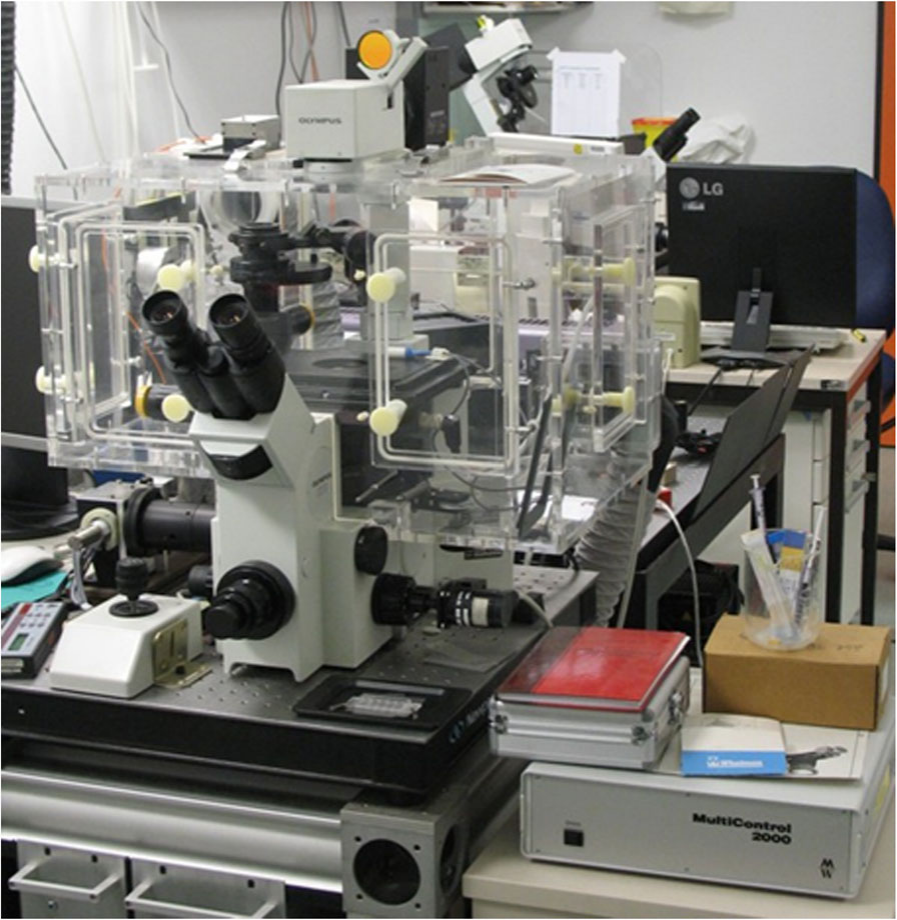
Fluctuation spectroscopy setup in Jörg’s group. The FCCS apparatus setup called FFM (Fluorescence Fluctuation Microscope), which is a combination of a confocal laser scanning microscope (CLSM) with a FCS module developed in Jörg’s group

In 1990 Jörg and Kati received an attractive offer from the Deutsches Krebsforschungszentrum (DKFZ) to put together a biophysics group and for Jörg to take a tenured professorship at the University of Heidelberg. These were the early 1990s, when not only whole Europe but even the realm of science was in turbulence, and at the DKFZ basic sciences - molecular- and cellular biophysics, molecular biology - were profoundly appreciated. So with the small group of people imported from Grenoble, the group started to work at the DKFZ. The construction of a new light scattering setup, the purchase of high edge spectrophotometers, an analytical ultracentrifuge (AUC) and an atomic force microscope (AFM) gave the experimental basis of the biophysics of macromolecules in 1994. Still focusing on the superhelical DNA conformation and DNA-protein interactions, Jörg’s know-how about flexible polymers was able to be extended to projects of other research groups. With Harald Hermann, who worked in the group of W. Franke, a neighbor on the 4th floor at that time, Jörg began to investigate the biophysical aspects of the intermediate filament (IF) oligomerization. That the fruits of this collaboration are still active, and that it produced 25 publications establishing the mechanism and kinetics of the oligomerization of vimentin and other IFs, is due to the commitment, among others, of Norbert Mücke. Norbert is one of the rare long-term members of Jörg’s group, and he has imparted his knowledge about analytical ultracentrifugation (AUC) and AFM to a moving stream of doctoral students. Groups in- and outside the DKFZ and Heidelberg have profited from these methods and have applied them from basic to biomedical research. Jörg had free energy to embark into new adventures, such as the construction of different fluorescence microscopy devices. It was in the air that the diffusion of fluorescently labeled molecules could be detected much more sensitively through the fluctuation of the fluorescent intensity than by light scattering. Based on the principle that less is more, fluorescence correlation or cross-correlation spectroscopy (Fig. 2) promised new perspectives.

As the precision of the commercial instruments was not satisfying, he designed and successfully constructed together with Michael Tewes a new microscope accessory, the patent of which was later purchased by Zeiss. The first FCS / FCCS results were obtained in a solution of chemically labeled DNA or DNA-protein complexes, but the interest turned quickly onto the intracellular interaction of macromolecules. The next development was facilitated generally by the expression of different fluorescent proteins and by the rigorous precision of Gabi Müller’s sample preparation. For the measurements of FCS in cells, the setup was further engineered by Malte Wachsmut to become a fluorescence fluctuation microscope (FFM). Jörg and doctoral student Nina Baudendistel together were able to quantitatively demonstrate for the first time the binding of two molecules to each other in live cells (Fos and Jun). From the slowed down mobility of the complexes, they could prove the binding of the heterodimers to chromatin. The potential of this FFM paved the way for different cellular applications, building long-lasting collaborations with French and Hungarian research groups.

The intracellular and intranuclear medium as playground for physical movements of the biomolecules also challenged the “simulators” of the Langowski’s group: a long line of postdocs and doctoral students (Christian Münkel, Gero Wedemann, Tobias Knoch, Frank Aumann, Annika Wedemeiet, Christian Fritsch, among others) who developed models for the 3 dimensional organization of the chromatin fibers, the chromosome territories and the permeability of the interchromatin space for small molecules. This last topic was supported by the FFM experiments of Nicolas Dross with GFP oligomers.

**Fig. 3:**
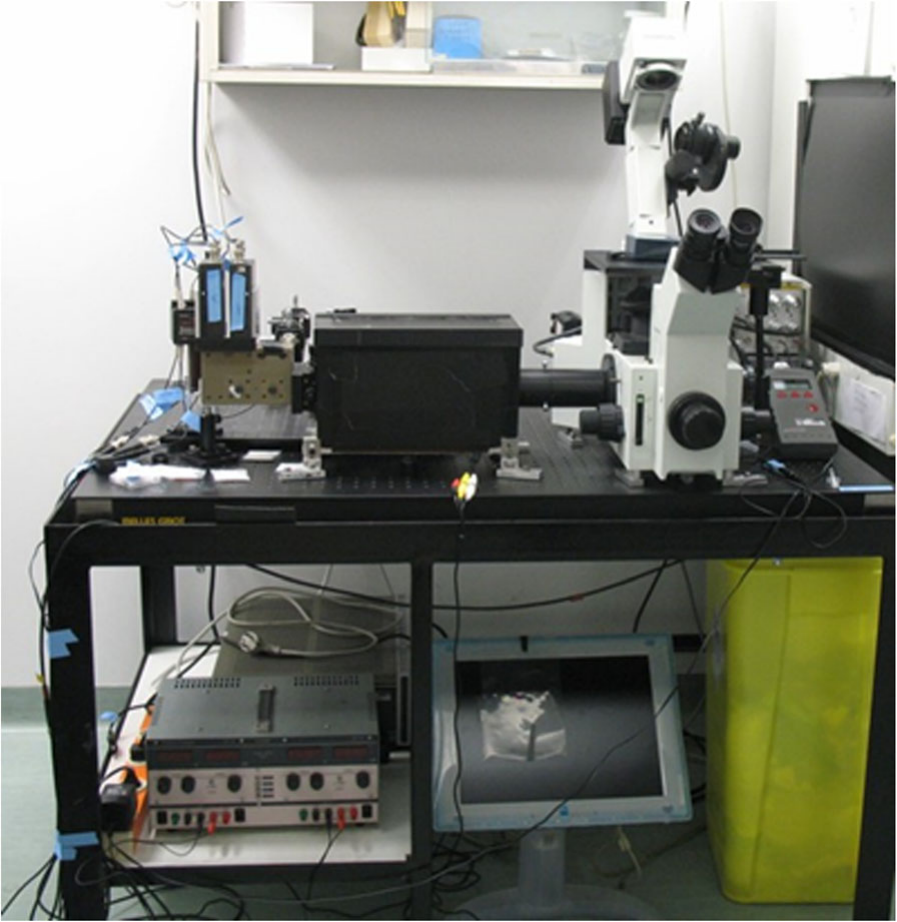
spFRET setup. The setup for spFRET developed in Jörg’s lab for the study of the assembly and disassembly processes of nucleosomes

In the new millennium the group moved to a new DKFZ building with more space and there Jörg contemplated the construction of a new microscope (Fig. 3).

**Fig. 4:**
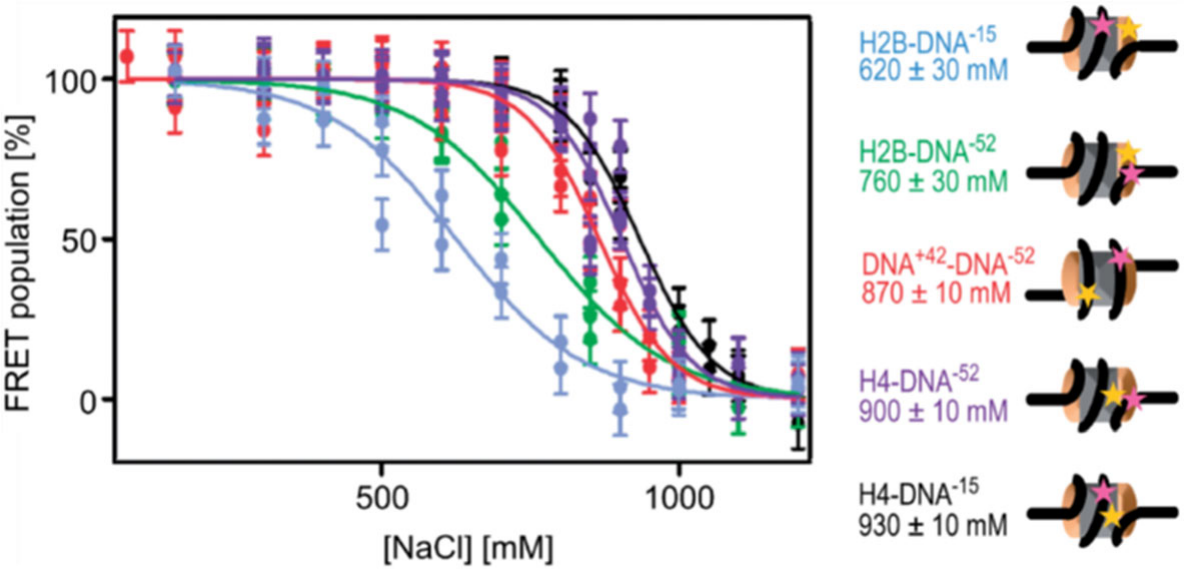
spFRET to study histones dissociation from the DNA. spFRET reveals an intermediate open conformation before H2A–H2B dimer dissociation from the DNA. Plot showing fraction of the FRET population as a function of the [NaCl] for H2B–DNA^− 15^ (blue), H2B–DNA^− 52^ (green), DNA^+ 42^–DNA^− 52^ (red), H4–DNA^− 52^ (violet) and H4–DNA^− 15^ (black). Each point represents an independent experiment. Cartoons of nucleosomes indicating the relative locations of labels on the nucleosome, together with the c_1/2_ denaturation values are also shown, using the same color scheme. Donor labels are shown in yellow, acceptor labels are shown in magenta. From the sequence of the loss of FRET between the different nucleosome subunits, a model for disassembly was derived (adapted from [75] with written permission given by the original license holders)

With Alexander Gansen (see his contribution later in text), then a doctoral student, they constructed a setup similar to the FCS, the single protein Foerster Resonance Energy Transfer (spFRET). By means of FCS one can observe the fluctuation of a few hundreds of molecules in the focus. In the spFRET method single molecules are examined one by one for their FRET signal, which enables one to measure distances between fluorescently labeled pairs on a particle in solution (Fig. 4). This new method, applied by Vera Böhm and Kathrin Lehmann in their theses, allowed one to learn more about the assembly and disassembly processes of nucleosomes, a key element in the accessibility of genes, and about the effects of histone modifications and mutations. Parallel to the spFRET technique, new simulation methods had to be developed to describe and predict the structure of nucleosomes, which are somewhat more complex than polymer-like DNA.

A series of doctoral students were involved in this development (Thomas Wocjan, Karine Voltz, Mithun Biswas, Ruihan Zhang) in close collaboration with Jeremy Smith (see his contribution later in the text), Jörg best friend from his time in Grenoble, then professor at the IWR. For the ultimate refinement of our spFRET studies, the group was helped by Claus Seidel from Düsseldorf, whose multiparameter setup was able to examine the fluorescence photons even closer and helps to identify dynamical processes. Basing on these results, the first concrete nucleosome structure could be easily created shortly after the appearance of affordable 3D printers, which Jörg quickly taught himself to use.

**Fig. 5:**
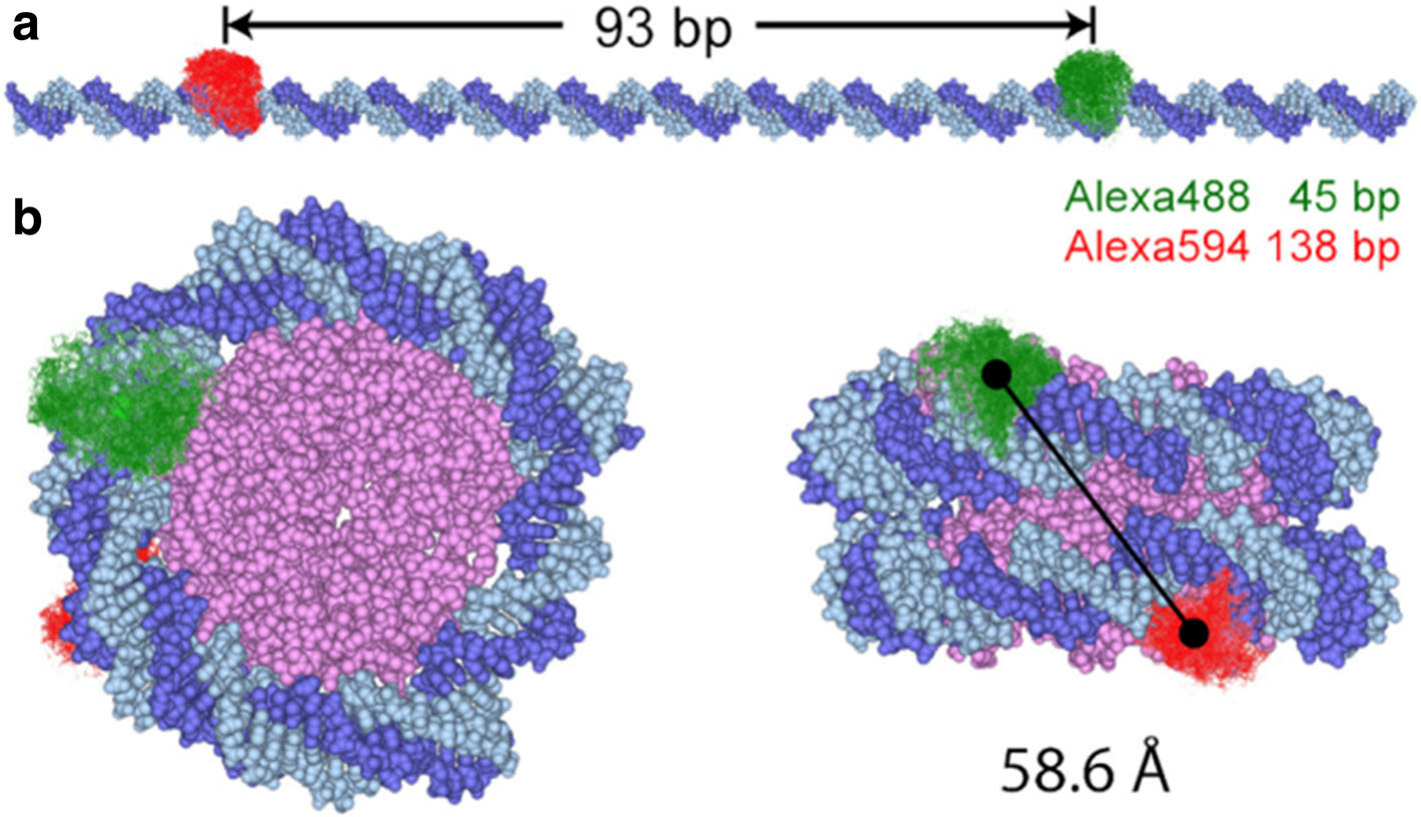
Molecular Dynamics simulations of nucleosomal DNA. Panel **a** Molecular Dynamics simulations of the conformational space of the D (green) and A (red) dyes in an extended nucleosomal DNA duplex [D strand (dark blue), A strand (light blue)]. Panel **b** Nucleosome viewed from top (Left) and from the side (Right), based on known crystal structures. Only the core of the histone (magenta) is shown for simplicity. The solid line connects the centers of mass of the fluorophores’ accessible space. Adapted from [28] with written permission given by the original license holders

**Fig. 6:**
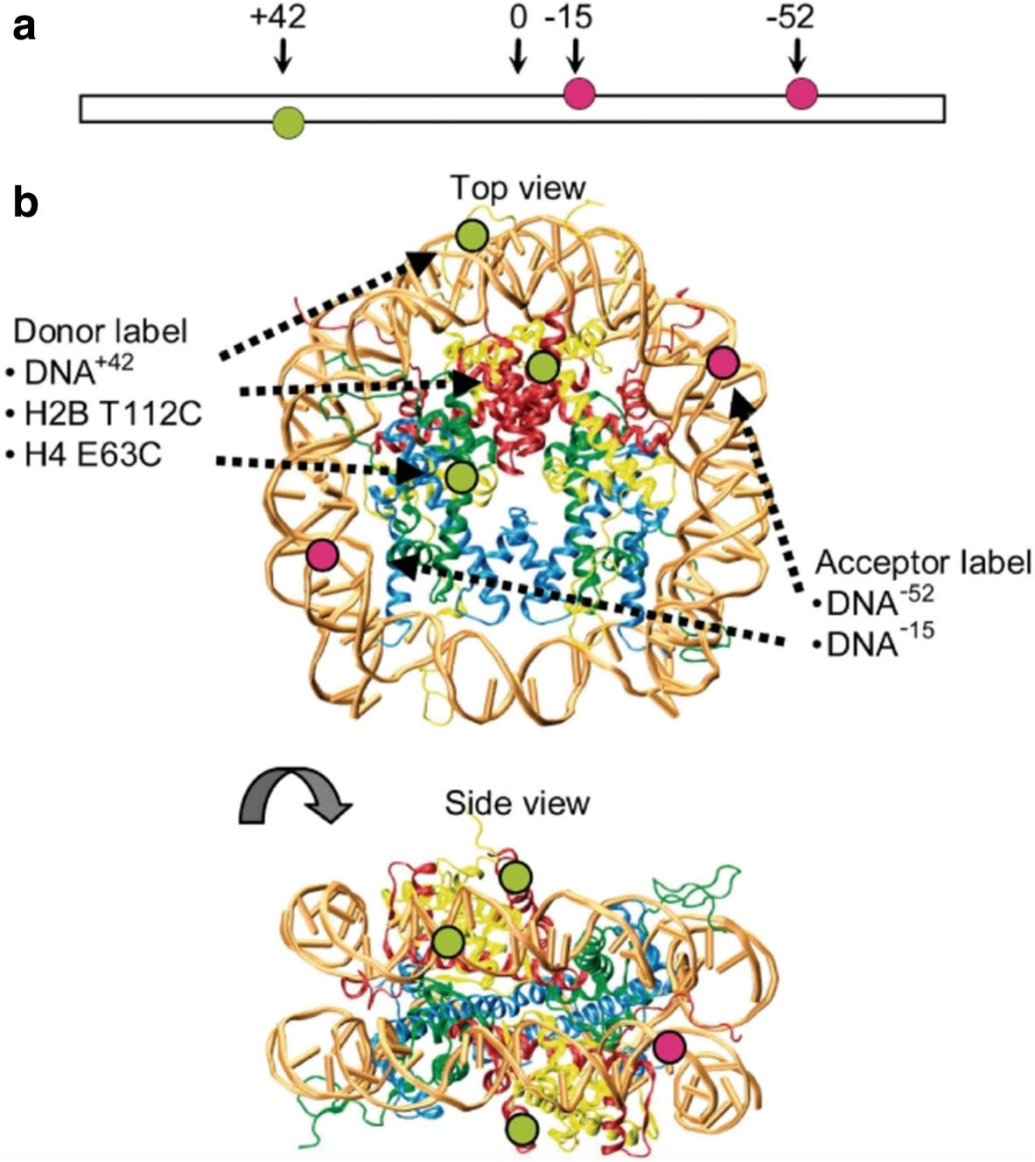
Fluorescently labeled nucleosomes used for FRET experiments. Panel **a** extended 170 bp nucleosomal DNA, the donor fluorophore Alexa 488 (green circles) and acceptor fluorophores Alexa 594 are shown in green and red, respectively. Panel **b** Top and side views of the nucleosome crystal structure: H2A is shown in yellow, H2B in red, H3 in blue, H4 in green. Adapted from [75] with written permission given by the original license holders

The wealth of experiments performed by the laboratory on different nucleosomal constructs prepared with single dye pairs for spFRET (Figs. 5, 6) brought to a great expansion over previous nucleosomal spFRET measurements [2]. The current and future direction of research in spFRET in the laboratory is following the effects of linker histones to the nucleosome structure.

**Fig. 7:**
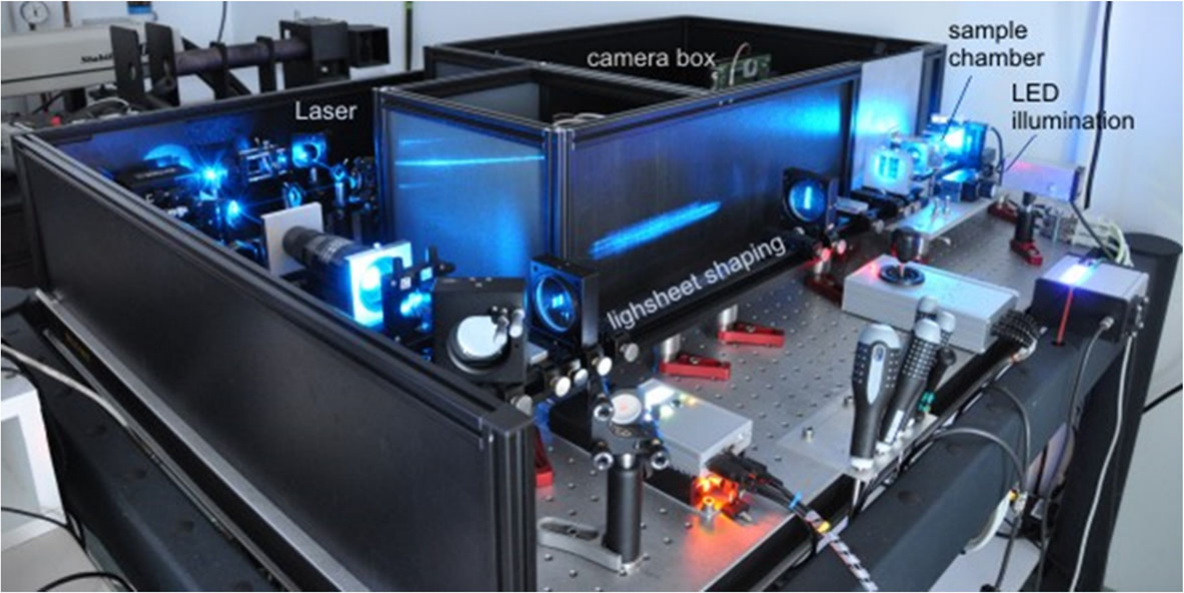
The Single Plane IIlumination Microscope- This setup was developed for the study of the dynamics in cells

In the next, his last, decade Jörg opened the way to a new technical development. Combining the Single Plane Illumination Microscopy (SPIM, Fig. 7) with quick sensitive fluorescence detection and profiting from Jan Krieger’s ceaseless activity, made it possible to construct and run a SPIM-FCCS setup [3]. This, like a light-tomograph, provides information about the 2 or 3 dimensional intracellular distribution, interactions and mobility of fluorescently labeled molecules inside individual living cells. With its help, one could map cells for studying the behavior of transcription factors, nuclear receptors and others. New types of questions can also be raised, and hopefully, answered, like the viscoelasticity of the cells and their nucleus, and the variations of the viscoelasticity between different cell states. The viscoelasticity is necessary for all transport and diffusive motions in the cell, and for these reasons Jörg started to look for appropriate methods to simulate it.

The further development of the SPIM setup is incomplete, due to two reasons: young scientists are forced to leave for industrial positions because even for the best ones, academic careers seem to be uncertain and frustrating; and because of Jörg’s unexpected passing. Worldwide there are only a few such instruments functioning yet and few people with the know-how.

The 3D or, with dynamical features, the 4D structure of the genome had fascinated Jörg for a long time, so with the appearance of the Hi-C results he jumped into this field and tried to combine them with his earlier simulations on nucleus organization. Soon he realized that only single cell results could shed some light on important questions while similarly to our microscopic methods, averaging kills the messages. Not the average, only the distribution of the behavior of single molecules or single cells yields real information. A long-time collaboration with Ada and Don Olins (see their contribution later in the text), having the same wide range of vision, exploited both the most classical biophysical methods, such as analytical ultracentrifugation, and the newest sequencing methods to study questions ranging from the peculiarities of the nucleosome surface until medically relevant gene-localizations in the nucleus.

## Methods

A wide variety of methods have been used in the research performed by Jörg Langowski or in collaboration with him. Apart from the general overview given in the Background section, different topics are reviewed and discussed more in detail in the following sections by some of his closest collaborators. We believe that the personal narrative of the colleagues, even with the unevenness of tones that personality brings with itself and the mixing of research considerations and personal remembrance, will offer a unique cross-sectional view of the research parabola that Jörg and his lab trailed over 30 years. Among others, these techniques include experimental approaches such as Atomic Force Microscope (AFM), Fluorescence Correlation Spectroscopy (FCS), Fluorescence Cross-correlation Spectroscopy (FCCS), Fluorescence Fluctuation Microscope (FFM), Photon Correlation Spectroscopy (PCS), Single Protein Foerster Resonance Energy Transfer (spFRET), Single Plane Illumination Microscopy (SPIM). Modeling and numerical approaches reviewed here comprise, among others, Brownian Dynamics (BD), Molecular Dynamics (MD) and Monte Carlo approaches.

Several systems have been investigated by these experimental and numerical methods, all at different degrees related to the structure and dynamics of chromatin in its different states.

## Results and discussion.

### Giuseppe Chirico: Twisting and jiggling like a DNA helix

In May 1989, I was visiting for the first time the laboratory of Jörg Langowski, just installed at the EMBL outstation in Grenoble. I was a PhD student in biophysics, looking for support in the preparation of supercoiled DNA to study its dynamics. I knew from the literature that chromatin structure and, most important its dynamics, was essential in a number of genetically relevant processes. At that time, Jörg was one of the leading scientists in this field. He was fond of the dynamics of the DNA helix and wanted to set up methods to understand its role. Now, I see how much this was really the key point, his major passion, beside family and gliding.

After the completion of my PhD, I joined Jörg in Grenoble and started, under his supervision and inspiration, to devise a model for the numerical simulation of the dynamics of DNA topoisomers. Jörg had published a number of pioneering experimental works on the study of the dynamics of the DNA helix in which he put much effort in studying highly purified and characterized samples. In most of the cases, he and his coworkers tried to get quantitative information from the analysis of the dynamics structure factor that was measured from the auto-correlation function of the scattered light. The analysis, though, was limited to the adoption of heuristic equations in analogy with polymer physics [4]. Our effort in the years 1990–1992 was to establish an exact, yet computationally accessible, mechano-elastic model of the DNA superhelix. We aimed first at the isolated macromolecule, moving later to (still crude) models of chromatin. Our calculations were based on the theory of dynamics light scattering and on the numerical algorithms for the Brownian dynamics of macromolecules.

#### In the lab

Photon Correlation Spectroscopy (PCS) is based on the use of coherent monochromatic light (wavelength *λ*) and on the observation of the dynamics of the speckle pattern arising from the superposition of a high (10^6^) number of scattering sources. A non trivial concept here is that the speckle pattern arises because of the superposition of the wavelets scattered by many independent sources, but the dynamics of the pattern at any point on the observation plane depends on the statistical properties of the single source dynamics: the process is based on the coherent properties of light. The spatial resolution over which we sample the dynamics is the reciprocal of the exchanged wave vector, ${\left|\overrightarrow{Q}\right|}^{-1}$ ~ λ/sin(θ/2), that decreases with the scattering angle, *θ*. Jörg, together with Mickey Schurr, was one of the first to exploit PCS to sample the internal motion of supercoiled DNA [5–7]. The molecular dynamics is over-damped. Therefore, we expect to characterize it by means of a spectrum of relaxation times and relative amplitudes [4]. We can single out the lowest frequencies out of this spectrum by measuring the correlation function G_I_(τ) of the scattered intensity $I\left(t,\overrightarrow{Q}\right)$ at the scattering vector $\overrightarrow{Q}$ In general, at least for non-pathological intramolecular dynamics, we can establish a general relation between this function and the statistics of the molecular displacement. For a small molecule (hydrodynamics radius R_G_ < < λ) for which we focus simply on the center of mass displacement, we write:

1 $$ {\displaystyle \begin{array}{c}{G}_I\left(\tau \right)={\left\langle I\left(t+\tau, \overrightarrow{Q}\right)I\left(t,\overrightarrow{Q}\right)\right\rangle}_t={\left\langle I\left(t,\left|\overrightarrow{Q}\right|\right)\right\rangle}_t^2\left(1+{f}_{coh}{\left|\int {d}^3{\overrightarrow{\Delta}}_0{e}^{i{\overrightarrow{\Delta}}_0\cdot \overrightarrow{Q}}P\left({\overrightarrow{\Delta}}_0\right)\right|}^2\right)\\ {}\propto {\left\langle I\left(t,\left|\overrightarrow{Q}\right|\right)\right\rangle}_t^2\left(1+{f}_{coh}\exp \left(-\frac{1}{3}{\left|\overrightarrow{Q}\right|}^2\left\langle {\left|{\overrightarrow{\Delta}}_0\right|}^2\right\rangle \right)\right)\end{array}} $$

In Eq. 1, ${\overrightarrow{\Delta}}_0=\overrightarrow{r}\left(t+\tau \right)-\overrightarrow{r}(t)$ is the translational displacement (center of mass) and f_coh_ is a factor that accounts for the degree of coherence of the collected field. If we are looking at a “complex” polymer, such as DNA molecules or DNA-protein complexes are, we describe it by its center of mass, ${\overrightarrow{R}}_{cm}$, and the displacements of the subunits from it. In this case, we have contributions from the translational diffusion of the center of mass (whose translational diffusion coefficient is D_T_) and the molecular “internal motions”. Altogether, this problem was approached with the help of a heuristic functional form for the intensity correlation function [7]:

2 $$ {\left\langle I\left(t+\tau, |\overrightarrow{Q}|\right)I\left(t,|\overrightarrow{Q}|\right)\right\rangle}_t\propto 1+{f}_{coh}{\left[\mathit{\exp}\left(-{\left|\overrightarrow{Q}\right|}^2{D}_T\tau \right)+ bexp\left(-\lambda \left({\left|\overrightarrow{Q}\right|}^2\right)\tau \right)\right]}^2 $$

In Eq. 2 the second, faster, relaxation rate, *λ*_*int*_, depends on the scattering vector and is typically fit by a linear trial function giving as the intercept the tumbling rotational diffusion and as a slope at high Q values, the so called *internal diffusion coefficient*, D_int_. The relative weight of the internal motion over the translational diffusion is measured by the factor “b” in Eq. 2 and it is a relevant parameter that has been explored experimentally as a function of the ionic strength. That analysis was applied in several studies of DNA molecules in vitro [8, 9] and it allowed us to bring into evidence the presence of DNA internal motions that were systematically faster in the supercoiled with respect to the relaxed circular DNA state [7].

However, that was not clearly the end of the story, and some groups resorted to numerical simulations [10, 11]. Langowski’s group was among the few ones that were systematically pursuing the comparison of the experimental output with more and more sophisticated models which were investigated by means of Monte Carlo [12] and Brownian Dynamics (BD) [13, 14] simulations. The role of the monovalent salts [8] and the topoisomers were first carefully studied experimentally [12] and then by numerical simulations and reported in a number of papers [15–17]. In total, these papers were an unprecedented effort to assess the DNA dynamics at a quantitative, molecular, level.

#### Numerical simulations never substitute experiments

Jörg was very fond of technology. As a young student, he was already programming on the Macintosh (Apple) computers, which, at that time, was the state of the art of personal computers. Many other technologies would come later, and Jörg kept up to date with them. Yet, he never jumped to the computational side as a whole. Experiments were always his leading research field, though he produced a huge literature based on numerical simulations, mainly Monte-Carlo and Brownian Dynamics, because he realized that the complex machinery of biology would never be addressed analytically in a satisfactory way. This second field was our common research when I joined his group in Grenoble (in 1990) and later in Heidelberg (in 1993). When looking back at his literature now, I find it astonishing as he pursued systematically his goal, by first building up methods, really new computational methodologies and not simply applications of existing ones. Our work on the simulation of the writhing of supercoiled DNA molecules (1200 base pairs long) was preceded by the setting and testing of a second order BD algorithm. The development of the mechano-elastic equations was performed in the framework of the Euler angles, starting from a bead of string model and adding a ribbon on each of the bonds [18]. Jörg’s intuition came first, preceding and guiding my subsequent development in differential geometry.

**Fig. 8:**
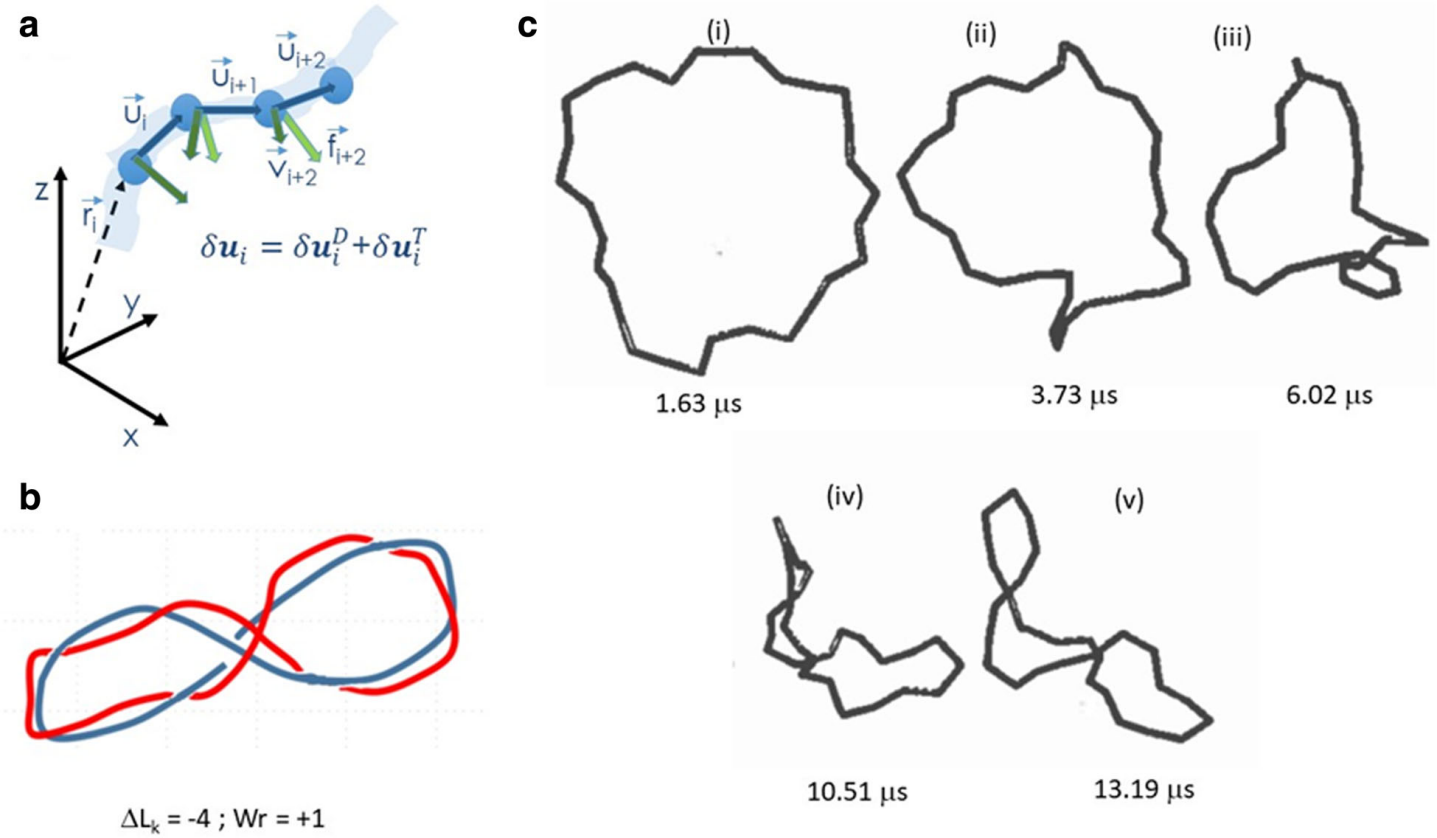
Mechanical model for the Brownian dynamics simulation of looped DNA supercoiling. Panel **a** string-of-beads model of N beads with coordinates {**r**_i_}_*i* = 1..N_. On each bead a local frame of reference {**f**_j_,**v**_j_,**u**_j_}_j = 1..N_ is set. The infinitesimal displacement of each bead is due to bending and torsional motion of the whole chain. Panel **b** sketch of a supercoiled plectonemical structure of linking number ΔLk = − 4. Panel **c** examples of the DNA structure simulated for ΔLk = − 4 at increasing simulation times as indicated in the figure (adapted from [18] with written permission given by the original license holders)

The aim was to tackle the writhing dynamics of an initially torsionally stressed, planar configuration of a circular molecule. This aim was already the subject of two seminal papers appeared in 1992 by Tamar Schlick and Wilma Olson who approached three-foil knotting [19] and supercoiling energetics [20] with a molecular dynamics approach based on a continuous B-spline modeling of DNA. These works were surely an inspiration for Jörg in setting up a Brownian dynamics model of supercoiling. Our approach was based on the computation of the virtual infinitesimal displacements set up by Allison et al. [21] and extend them to the case of a potential function that depends both on the translational and rotational degree of freedoms. The DNA chain was described by a string of beads (with coordinates {**r**_i_}_*i* = 1..N_) over which we superimposed a series of ribbons (whose orientation was described by the Euler angles {α_i_, β_i_, γ_i_}_i = 1..N-2_) joining the centers of the beads: translational motions were ruled by the diffusion of the beads and torsional motions were accounted for by the torsion of the ribbons (Fig. 8).

The derivation of the model was a story in its own. We started from an intuitive description that came organizing during long afternoon discussion with Jörg. I was spoiled by analytical mechanics courses at the university, Jörg was seeing molecules crimpling and inter-wining under torsional stress. We submitted a paper and the reviewer happened to be Mickey Schurr who met us in the French Alps during a meeting; we ended up sitting for two days in a bar trying without success to write an analytical model in Mickey’s yellowish notebook. However, this bar experience helped us a lot and after a few weeks, we developed a reasonable approach, based on the infinitesimal displacements of each of the beads of the string-of-beads model for either a pure translation or a pure rotation:

3 $$ {\displaystyle \begin{array}{l}\left\{\begin{array}{c}{b}_i{\delta}^D{\mathbf{u}}_i=\delta {\mathbf{r}}_{i+1}-\delta {\mathbf{r}}_i+{\mathbf{u}}_i\left({\mathbf{u}}_i\cdot \delta {\mathbf{r}}_i\right)-{\mathbf{u}}_i\left({\mathbf{u}}_i\cdot \delta {\mathbf{r}}_{i+1}\right)\\ {}{\delta}^D{\mathbf{f}}_i=-{\mathbf{u}}_i\left({\mathbf{f}}_i\cdot {\delta}^D{\mathbf{u}}_i\right)\\ {}{\delta}^D{\mathbf{v}}_i={\delta}^D{\mathbf{u}}_i\times {\mathbf{f}}_i\end{array}\right.\\ {}\left\{\begin{array}{c}{\delta}^T{\mathbf{u}}_i=0\\ {}{\delta}^T{\mathbf{f}}_i={\delta \phi}_i\left({\mathbf{u}}_i\times {\mathbf{f}}_i\right)\\ {}{\delta}^T{\mathbf{v}}_i={\delta}^D{\phi}_i\left({\mathbf{u}}_i\times {\mathbf{v}}_i\right)\end{array}\right.\end{array}} $$

where the torsional angle was related to the Euler angels by *δ*( *α*_*i*_ + *γ*_*i*_ ) = *δ ϕ*_*i* + 1_ − *δ ϕ*_*i*_. From these and the potential energy, that was a collection of bending and torsional springs, we derived the forces and torques of each bead. The derivatives of the potential function with respect to the translational or the rotational coordinates provided, among other conventional terms, a mixed term, the so-called torsional force, that was arising from the change in the torsional component of the energy with respect to the translational coordinates [22]. It was then essential to close the molecule on itself by setting a linking number ΔL_k_ as

4 $$ \left\{\begin{array}{c}{\beta}_i=-2\pi /N\\ {}{\alpha}_i=-\left(i-1\right)2\pi \varDelta {L}_k/N\\ {}{\gamma}_i=i2\pi \varDelta {L}_k/N\end{array}\right. $$

and to plug all this in a coupled translational-rotational BD algorithm [18]. This supercoiled model was then applied to the study of the effect of salt (ionic strengths) and intrinsically curved structures inside the plasmid [16]. Both topics were highly relevant at that time indicating, as often happened, how Jörg was always problem oriented.

Jörg and I discussed sometimes about the difference in our approaches. His view was sometimes difficult for me to share, since Jörg was definitely not the “average biochemist”. Physicists, some of them at least, are more prone to theory and less to applications whereas biologists and biochemists know that we have to fight to survive and want to know how we work, as thermodynamic machines, to reach this goal.

**Fig. 9:**
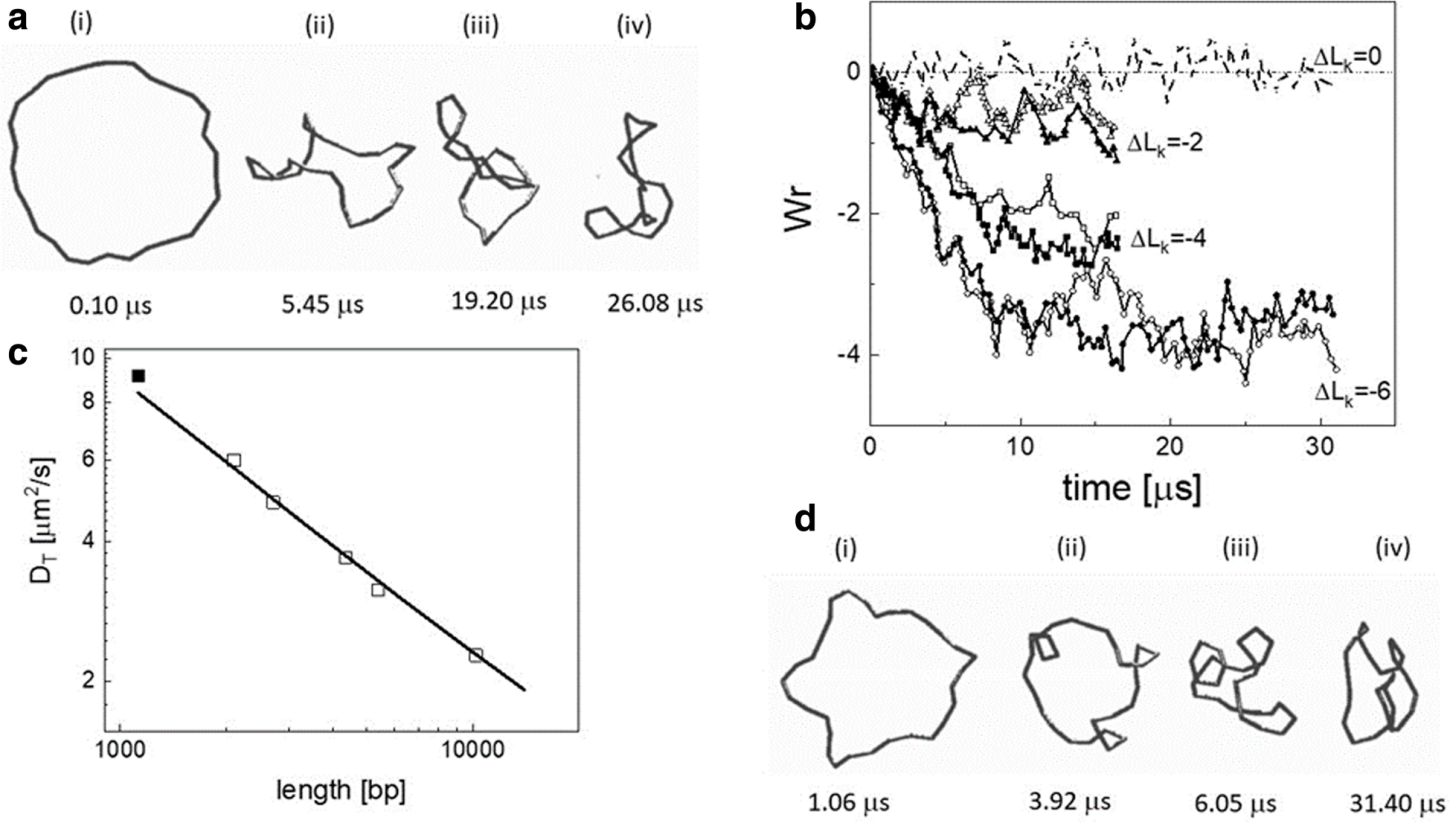
Brownian Dynamics simulations of supercoiled DNAs. Application of the BD algorithm for a supercoiled structure as a function of the linking number and the length. Panel **a** samples of the structures simulated for ΔLk = − 6. Panel **b** time evolution of the writhe of supercoiled structures sampled form different simulations starting from writhing number = 0 for ΔLk = 0 (dot-dashed), − 2 (triangles), − 4 (squares) and − 6 (circles). Panel **c**: translational diffusion coefficients as a function of the DNA length for ΔLk = − 4 measured (open squared) by Langowski, Giesen and Lehman [76] and simulated (filled square) by Chirico and Langowski [18]. Adapted from [18] with written permission given by the original license holders

In summary, this effort allowed us to follow in real time the writhing of supercoiled molecules of increasing L_k_ (Fig. 9a). From these visually astonishing (at that time) results, we could obtain translational diffusion values that scaled with the contour length of supercoiled plasmid DNAs (Fig. 9c). An additional output was the possibility to measure the kinetics of the writhing, which was further pursued by T. Schlick’s group in 1998 [23]. In the late 1990’s, the possibility to measure was much ahead of the technical possibility [24, 25]. Through single molecule FRET pairs or FCS experiments, these measurements are feasible nowadays. However, already those first simulations would not have been possible without the human and research support and inspiration I received from Jörg.

### Jeremy smith: Understanding nucleosome repositioning and dynamic histone tail function

I met Jörg for the first time in 1989 when he became a Group Leader at EMBL, Grenoble. Jörg was a young biophysicist in an organization that was trending more and more towards experimental biology. We were among the few in the organization who liked to discuss biological systems from the physical viewpoint. Although I soon left for Harvard and then Saclay, Jörg was pivotal in bringing me to Heidelberg in 1998 to what was the first Chair in Computational Biology in a German university. We also had personal involvement through the French elementary school in Heidelberg that Jörg helped found and where I sent my daughter, Serena. Professionally we organized and taught biophysics courses in the university together over the next 8 years. We also served together on several committees at Heidelberg University. We established a research collaboration in which simulation techniques from my lab were applied to chromatin problems brought by Jörg lab. This collaboration was effected by some talented graduate students, including Karin Voltz and Mithun Biswas as well as a postdoc, Jochen Erler.

Our collaboration involved using models of various resolutions: from analytical physics through to all-atom simulations. An initial interest was in the mechanism of nucleosome repositioning, a fundamental process in gene function. DNA elasticity is a key element of loop-mediated nucleosome repositioning. Two analytical physics models for DNA elasticity had been proposed: the linear sub-elastic chain (SEC), which allows DNA kinking, and the Worm-Like Chain (WLC), with a harmonic bending potential. In vitro studies had shown that nucleosomes reposition in a discontiguous manner on a segment of DNA and this had also been found in ground-state calculations with the WLC analytical model. Together with Biswas, we used Monte Carlo simulation of the dynamics of DNA loop-mediated nucleosome repositioning at physiological temperatures using the SEC and WLC potentials [26]. At thermal energies both models predict nearest neighbor repositioning of nucleosomes on DNA, in contrast to the repositioning in jumps observed in experiments. This suggested a crucial role of DNA sequence in nucleosome repositioning.

Histone tails play an important role in gene transcription and expression and Jörg’s group and mine also aimed to understand the structural and dynamic properties of histone tails in the nucleosome. We used for this purpose mainly molecular dynamics simulations. One of the results of this collaboration was the finding that there is a strong dependence on the force field in predicting the interactions between the histones and the DNA [27]. However, by using cluster analysis, we also found a single dominant configuration of binding to DNA for the H4 and H2A histone tails, whereas H3 and H2B show multiple binding configurations with an equal probability.

Results from both implicit and explicit solvent simulation models showed that large portions of the histone tails are not bound to DNA, supporting the complex role of these tails in gene transcription and expression and making them possible candidates for binding sites of transcription factors, enzymes and other proteins.

**Fig. 10:**
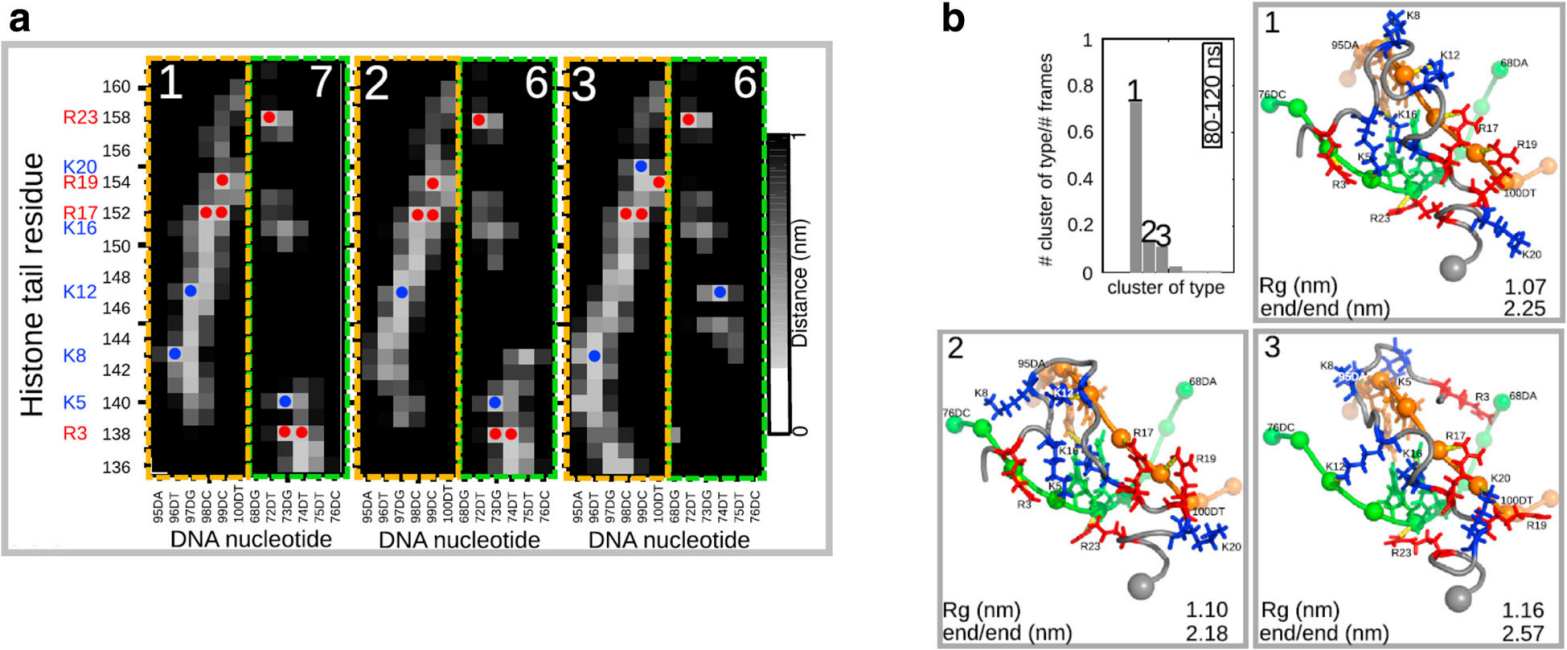
Analysis of the DNA-histones interactions. Panel **a** Key tail/DNA interactions of the histone H4 tail. Distance map of tail residues and the phosphate group of each nucleotide for the three most populated clusters of the H4 tail. Orange and green boxes indicate the two strands of DNA. Blue circles and red circles indicate contacts between lysines and arginines, respectively, and DNA phosphate (cutoff 0.3 nm). Green numbers denote the number of neutralized charges. Panel **b** Results of a cluster analysis of the H4 tail trajectory. Probability and conformations of the cluster representative of the three most populated clusters are shown. The cutoff was 0.2 nm and the last part of the trajectory (80–120 ns) was used. The two DNA strands involved in binding of the H4 tail are colored orange and green. Spheres represent phosphate groups of DNA, and lysine and arginine residues are colored blue and red, respectively. The gray sphere is the end of the tail connected to the histone core. Yellow lines indicate interactions between the histone tail and the DNA. Radius of gyration, Rg, and end-to-end distance are given for each cluster (adapted from [27] with written permission given by the original license holders)

Results from contact maps, distance maps, and cluster analysis (Fig. 10) showed that lysine and arginine residues make specific contacts with DNA. The positively charged side chains of histone tails were found at negatively charged phosphate groups or in the minor or major groove of the DNA. Using cluster analysis, the teams found dominant binding conformations for H4 and H2A, whereas H3 and H2B showed a broader probability distribution.

The 2014 paper [27] ends with an invitation to obtain experimental input from a high-resolution optical spectroscopy, FRET. Indeed Jörg’s group devoted much effort to study this issue, before and after the Molecular Dynamics (MD) simulation work with us. The seminal work in this field appeared with the names, among others, of Alex Gansen, Katalin Tóth and Claus Seidel [28] and the most recent efforts in this sense just appeared in 2017 [29], after Jörg’s passing. In the 2009 paper, single molecule FRET was employed to systematically characterize nucleosomes disassembly intermediates. 3 species were identified and assigned to structures, each with a different FRET response: (i) the most stable high-FRET species corresponding to the intact nucleosome, (ii) a less stable mid-FRET species ascribed to a first, partially unwrapped, DNA intermediate and less histones, and (iii) a low-FRET species characterized by a very broad FRET distribution, representing highly unwrapped structures and free DNA. Selective fluorescence correlation spectroscopy analysis indicated that even in the low-FRET state, some histones are still bound to the DNA. Moreover, a geometric model of the DNA unwinding was proposed, that could predict the presence of the observed FRET species.

This picture had a strong dynamic flavor. It is therefore quite expected that the work of both our groups and other teams was not limited to the structures and to mapping the histones-DNA interactions; we were also interested in simulating the dynamics, particularly that involved in the DNA detaching from the histones, which can be a trigger and/or a modulator for the DNA replication.

Our previous work [30] reported nanosecond regime (100 ns all-atom) MD and focused on the structures of the tails of the H2-H4 histones. These simulations suggested that the changed interaction on H3 tail removal is a result of a variation in the electrostatic potential at the H2A α3 domain induced by H3 tail clipping. Thus, the electrostatic potential at the H2A α3 domain may be a determining factor for nucleosome stability. Later this was tested [29] by introducing charge-modifying mutations that alter the electrostatic potential at the H2A α3 domain without clipping of the H3 tail. Jörg ‘s group designed two sets of mutated, recombinant *Xenopus laevis* H2A histones. In the first set, the positively charged arginine(s) are exchanged with neutrally charged alanine(s), while the second type incorporates a negative charge by exchanging arginine with glutamic acid. Examination of these constructs was found to fully support the 2012 simulation results.

**Fig. 11:**
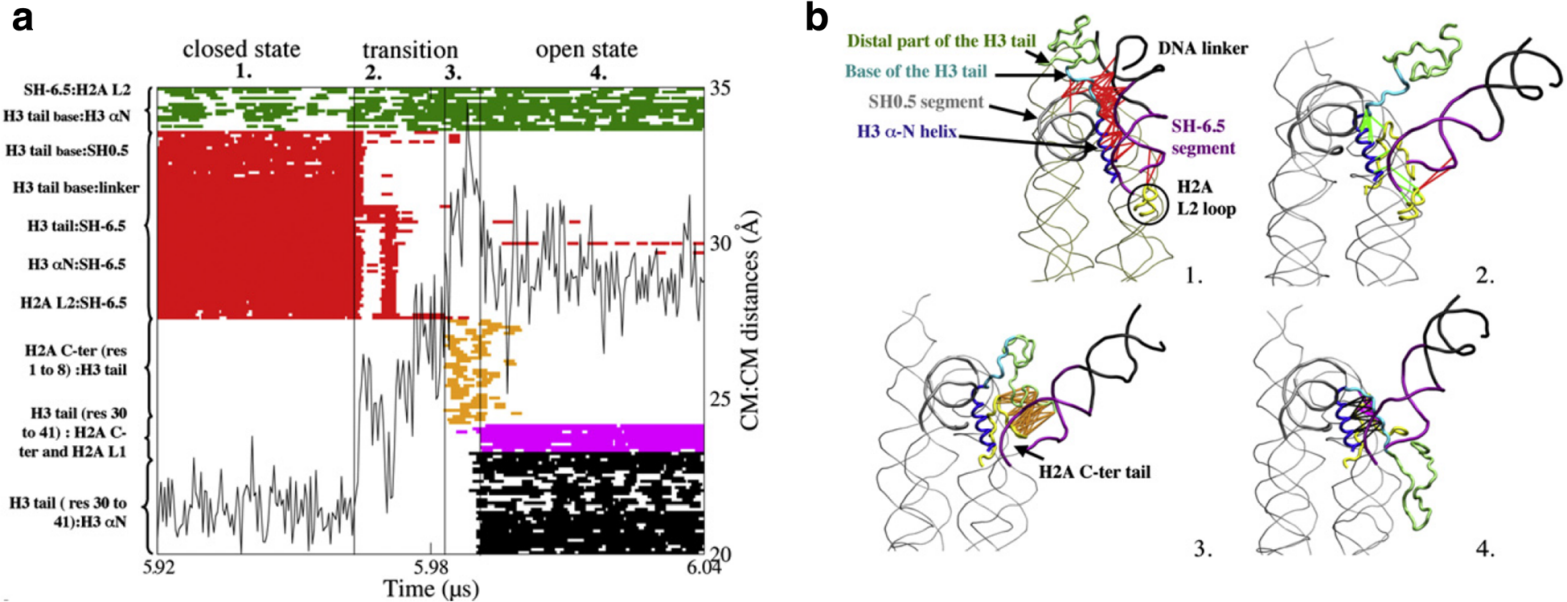
Open and closed states in the DNA-nucleosomes interactions. Panel **a** Formed and broken contacts in the CG structure of the nucleosome and H3 helix:SH-6.5 distance during the period encompassing the transition from the closed to the long-lived open state in simulation 1. (a1) Closed state: a tick is displayed when a native or a new-formed interaction is present. (a2) First phase of the transition: contacts broken and formed in the first phase are the same as for the short-lived open state. (a3) Second phase of the transition: contacts are formed between the extremity of the H3 tail and the extremity of the C-terminal tail of H2A. (a4) Open state: at the end of the transition phase, the H3 tail forms new contacts with the H3 aN helix and with the C-terminal extension and the L2 loop of H2A. Interactions of the distal part of H3 are not displayed. Panel **b** Representation of the nucleosome in (b1) the closed state, (b2) the first and (b3) the second phases of the transition state, and (b4) in the long-lived open state. The lines correspond to the interactions displayed in a. adapted from [31] with written permission given by the original license holders)

The focus in a 2012 paper was the histone H3 [31] The simulations were performed with a coarse grained model that allowed to simulate microseconds dynamics. In order to perform the coarse grained work, we needed to develop a force field, and this was performed using self-consistent multiscaling [32]. The longer, coarse-grained simulations reported in the 2012 paper [31] showed short-lived, reversible DNA detachments from the nucleosome and long-lived DNA detachments not reversible on the timescale of the simulation. During the short-lived DNA detachments, 9 bp dissociate at one extremity of the nucleosome core and the H3 tail occupies the space freed by the detached DNA. The major result was that the long-lived DNA detachments are characterized by structural rearrangements of the H3 tail including the formation of a turn-like structure at the base of the tail that sterically impedes the rewrapping of DNA on the nucleosome surface. This result is a strong hint to the role played by the DNA-histone dynamics in the histone regulation activity, a fact that motivated a whole set of papers. Again, in this paper one of the signatures of the cooperation between us appeared in the elaborate and effective display of the results of the numerical simulations, as can be seen in Fig. 11, adapted from [31].

### Ada L. Olins and Donald E. Olins: Chemical bonds.

Jörg was definitely not a cell biologist; we are clearly not biophysicists. So, what was the bond between us? What provoked us to return to Jörg and Kati’s lab for three months every year, for 6 years, from 2012 to 2017? More on this question later. First, an anecdote, which happened repeatedly in various forms during our annual visits.

We would march into Jörg and Kati’s office, saying: “We want to show you some exciting immunostaining images that we just obtained.” During our exposition, we would notice Jörg’s eyes becoming “glassy”. Suddenly, he would shout: “I don’t understand a word that you are saying! I don’t understand this stuff. Let me tell you something really interesting! Active Brownian motion.” He would begin his description and gradually our eyes would become “glassy”.

So, what was the bond between us? We were each enthusiastic about our own science, and able to see that enthusiasm in the other person. Jörg and Kati were extremely generous with their laboratory resources and their time. We had a bench in their lab, and often received the advice and assistance from their talented technical staff. We were FREE to explore our own ideas. Jörg and Kati were definitely interested in our experiments and tutored us in the use of their equipment. Jörg helped us in all things “computer-related”, while we listened to his diatribes against Bill Gates. Jörg, like us, was adamant about scientific truth and personal honesty. We truly felt that we were kindred spirits.

Jörg was an extremely talented person. He spoke many languages, organized the scientist’s march in Heidelberg, was an ardent runner, a fabulous cook, a supporter of liberal politics and full of joy for life. His friendship was a total pleasure.

The major scientific issue that we had in common with Jörg and Kati was the perspective of the polymer properties of chromatin. We, in particular, have been fascinated by the diversity of chromatin polymer conformations reflecting the various structural and functional states. Just consider some (not an exhaustive list) of the described structural and functional chromatin states: nuclear envelope association; epichromatin; Lamina Associated Domains (LADs); heterochromatin; euchromatin; interphase chromosome territories; mitotic chromosomes; chromomeres; TADs (Contact Domains, Compact Domains); polytene chromosomes; lampbrush chromosomes; synaptonemal complexes etc., etc. All of these chromatin states (and more) build upon the nucleosome [33]. Jörg and Kati were deeply interested in the conformational states of the mononucleosomes. They have studied these states under many conditions in very creative experiments.

During our brief annual visits to the Langowski group, we utilized our intellectual freedom to pursue projects that also involved new collaborators outside the DKFZ, often based upon introductions to Jörg’s friends and colleagues. We discussed these experimental directions with Jörg and Kati. All of the projects involved analysis of features of “our favorite cell”, HL-60/S4. This cell line is derived from a human female, who died from Acute Myeloid Leukemia (AML). The key advantage of this cell line is that it can be differentiated quickly (several days) in vitro with simple chemical additives: into granulocytes (using Retinoic Acid, RA); monocytes (using vitamin D3); or macrophage (using phorbol ester). During differentiation with RA, the nuclei change shape, becoming lobulated, exhibiting extensive nuclear envelope growth and formation of Nuclear Envelope-Limited Chromatin Sheets (ELCS) [34]. Employing electron microscopic tomography on plastic-embedded and stained HL-60/S4 granulocytes with collaborators in Colorado and at EMBL (Heidelberg), we had observed that the chromatin of ELCS (previously, thought to be a single sheet of 30 nm diameter chromatin fibers sandwiched between two apposed nuclear envelopes, which are separated by ~ 30 nm), in fact revealed a criss-cross pattern of two layers of ~ 15 nm irregular fibers. Companion cryo-electron microscopy on ELCS clearly indicated that the distance between the apposed nuclear envelopes is ~ 60 nm in the vitrified ice, implying ~ 50% shrinkage during fixation and plastic embedding of the ELCS [35]. In the hydrated state, the chromatin fibers appear to be stretched in parallel arrays to their closest inner nuclear membrane displaying a non-uniform diameter of ~ 30 nm.

From other studies, prior to joining the Langowski lab, we had shown that certain mouse monoclonal antibodies, including PL2–6, yielded strong immunostaining at the surface of chromatin adjacent to the nuclear envelope and at the surface of mitotic chromosomes (regions denoted, “epichromatin”) [36]). While in the Langowski lab, we embarked on characterizing interphase epichromatin, specifically defining the epichromatin DNA sequence elements present in undifferentiated and differentiated forms of HL-60/S4 cells. We developed a new type of ChIP-Seq, in order to enrich epichromatin [37]). Some of the surprising results of the ChIP-Seq were: 1) Only ~ 4% of the genome is represented within the epichromatin of undifferentiated and differentiated cell forms. 2) Retrotransposon Alu is enriched ~ 10-fold within HL-60/S4 epichromatin, compared to the average nuclear content of Alu. 3) The mapped distribution of epichromatin along human chromosomes is discontinuous (i.e., alternating epichromatin enriched and depleted regions), with a conserved pattern comparing the undifferentiated and differentiated cell states.

In the years of our visits, we also collaborated on studies with Jörg, that described the transcriptomes of HL-60/S4 undifferentiated and differentiated cell states [38], defined nucleosome positioning and repositioning in the various cell states [39] and identified the DNA methylation patterns within the various cell states (manuscript in preparation).

Most recently, while in the Langowski lab, we have come closer to a molecular definition of the epichromatin epitope [40]. We currently suspect that PL2–6 is binding to the nucleosome acidic patch, implying that this epitope is “exposed” at the chromatin surface adjacent to the nuclear envelope. One other feature has emerged from this most recent study. PL2–6 is a *bivalent* antibody which generates the characteristic epichromatin surface staining pattern. However, after papain digestion of PL2–6 and the preparation of *monovalent* Fab fragments, the immunostaining pattern changes dramatically. On fixed and Fab-immunostained HL-60/S4 cells, chromatin surface staining is lost; instead, a “punctate” staining pattern is noted throughout interphase nuclei and along the arms of mitotic chromosomes. These stained spots, named “chromomeres”, are ~ 200–300 nm in diameter and are estimated to be several thousand per interphase nucleus or mitotic chromosome set. The identity of chromomeres is currently under investigation; one prime candidate is the “compact domain”, that contain ~ 600–1000 nucleosomes in a chromatin liquid droplet [41]).

Jörg Langowski and Kati Tóth have been wonderful hosts for our brief visits to their lab. They encouraged our studies and participated in discussions and criticisms. They introduced us to other collaborators (Vladimir Teif, who analyzed the nucleosome positioning [39], and Justin O’Sullivan, who has conducted Hi-C experiments on the HL-60/S4 cells {submitted for publication}). Jörg carried the torch for Open and Honest scientific studies. He has spawned many new torch bearers.

### Alexander Gansen: Nucleosomes and single-protein fluorescence resonance energy transfer (spFRET)

As we saw from the previous paragraphs, Jörg Langowski devoted his scientific life to the analysis of the structural and functional properties of DNA and chromatin – the hierarchical compaction of DNA inside eukaryotic cells. Fascinated by the question, how the genetic material can be condensed into a nucleus of less than 10 μm in diameter, while providing rapid on-demand access to specific DNA loci when needed, he approached this complex structure from experimental as well as theoretical perspectives. He did not focus on a particular aspect of chromatin per se, but rather probed chromatin properties on different length scales, ranging from chromatin morphology and its impact on protein mobility in whole cell nuclei to structural properties of chromatin on the sub-nanometer scale. Over the years he established and refined several biophysical methods in his lab, from ensemble techniques such as Photon Correlation Spectroscopy (PCS) and Fluorescence Correlation Spectroscopy (FCS) to single molecule methods like Atomic Force Microscopy (AFM) and single-protein FRET (spFRET) spectroscopy. In this section, I will summarize Jörg’s major accomplishments on nucleosome structure and dynamics, particularly those related to the use of spFRET spectroscopy.

At the molecular level the central component in chromatin is the nucleosome, which consists of roughly 150 bp DNA wrapped almost twice around 8 histone proteins (2 copies each of H2A, H2B, H3 and H4 or their variants). In vivo, nucleosomes are connected by short stretches (10–90 bp) of linker DNA to form a bead-on-a-string structure, which condenses with the help of linker histones into higher-order structures, the exact conformation of which is still under debate. In recent years it became evident that changes in nucleosome structure affect all levels of chromatin organization – from local nucleosome-nucleosome interactions to interactions between global chromatin domains [42] - and thus have immense impact on genetic activity. Dedicated protein machineries can alter nucleosome structure and stability to facilitate temporary access to DNA by repositioning of nucleosomes along DNA, re- and disassembly nucleosomes during transcription or chemically modify the nucleosome surface.

As a physico-chemist with long-time expertise in DNA topology, Jörg became attracted to this complex structure and its biological importance. In the late 1990’s several high resolution X-ray structures of nucleosomes provided atomic details of the arrangement of DNA and histone proteins within the complex; however, the dynamic behavior of nucleosomes under near physiological conditions remained elusive. Having established a new biophysical research lab at the German Cancer Research Center, Jörg and his group embarked on an experimental quest to unveil the structural and dynamic properties of nucleosomes in more detail.

The method of choice for these studies when I joined the group became fluorescence spectroscopy – in particular Förster Resonance Energy Transfer (FRET), a technique that can probe the dynamic properties of individual nucleosomes and small nucleosome arrays with high spatial and temporal resolution. FRET is a non-radiative energy transfer between a donor and an acceptor fluorophore as they approach each other to within a few nanometers, resulting in a measurable decrease in donor fluorescence intensity and lifetime and an increase in acceptor signal. The extent of energy transfer scales with the inverse 6th power of the interdye distance and provides a “molecular ruler” on the nanometer scale that is well suited to study dynamic processes within nucleosomes. Changes in nucleosome architecture can be directly followed via changes in the FRET signal. In the ‘90s, the Langowski lab implemented a variety of different FRET strategies that were tailored to address nucleosome dynamics and other DNA-protein interactions. Single molecule FRET was the next step ready to be setup when I joined the group.

#### The early days – Mapping the linker DNA geometry in nucleosomes

Between 1998 and 2001 Jörg and Kati Toth, his wife and scientific collaborator, performed a series of bulk FRET experiments to map the geometry of DNA entering and exiting the nucleosome. By reconstituting nucleosomes with different length of DNA and labeling their ends with Fluorescein and Rhodamine X they could show that in solution the linker arms assume a more open conformation than in the crystal, and that addition of histone H1 decreased the distance between linker DNA ends [43]. Chemical acetylation of histone tails modified the entry-exit geometry – while acetylation of H4 was found to have no effect on opening, acetylation of histone H3 opens up the nucleosome ends, linking its physiological role as an epigenetic marker for gene activation to increased chromatin accessibility through changes in nucleosome architecture. This work was among the first to apply bulk FRET to questions related to the structural properties of nucleosomes.

In vivo, nucleosomes are not isolated but part of a complex network, where the presence of neighboring nucleosomes could significantly modulate nucleosome properties. Following their initial work on mononucleosomes, bulk FRET was then combined with atomic force microscopy to study trinucleosome arrays reconstituted on 600 bp long DNA. Data suggested that the structural changes due to salt concentration, acetylation and linker histone binding are similar to those of mononucleosomes [44] – results, that were later corroborated by the work of Poirier et al. [45], who showed by a combination of FRET and Fluorescence Correlation Spectroscopy (FCS) that the rate of DNA unwrapping and protein binding to buried nucleosomal DNA is not significantly altered by the presence of flanking nucleosomes on either side.

#### The next step – Establishing single molecule FRET spectroscopy

Despite the wealth of information that could be obtained with bulk experiments, it soon became clear that more sophisticated experiments were needed to address nucleosome architecture and dynamics in detail. With the advent of appropriate soft- and hardware tools nucleosome architecture could now be probed one molecule at a time. This way, the structural heterogeneity within the ensemble can be resolved – information that is averaged out in bulk experiments – including the presence and lifetimes of short-lived intermediate states and kinetic rates of their interconversions.

Complementary to the efforts of Sanford Leuba’s group and John VanNoort’s lab, who both established a framework for single protein FRET (spFRET) on immobilized nucleosomes, Jörg’s lab focused on establishing spFRET on freely diffusing nucleosomes – here, Jörg’s more than 20 years expertise with light scattering and FCS came in handy as both methods require similar instrumentation to study molecules in solution.

In diffusion-based spFRET samples are diluted to several tens of pM to ensure that nucleosomes move though a laser focus one-at-a-time. The laser focus is typically generated by a confocal microscope. Individual bursts of fluorescence can be discriminated against the background, and FRET efficiencies (and apparent interdye distances) can be computed for each molecule. The distribution function from many of these molecules can reveal subpopulations in a heterogeneous ensemble.

While the sensitivity necessary to discriminate individual molecules was readily achieved using state-of-the-art optical components the low concentrations required for single molecule identification posed a problem: if nucleosomes are to be studied under near-equilibrium conditions, sufficient sample integrity is required at concentrations, where nucleosomes are usually no longer stable. For once, adsorption of nucleosomes to the container walls had to be minimized. Second, spontaneous nucleosome dissociation has to be prevented otherwise no meaningful data can be obtained during the time needed for the experiment. Our first spFRET paper hence focused on various surface passivation and nucleosome stabilization strategies, such as adding excess inert protein or unlabeled nucleosomes into solution, which laid the ground work for more sophisticated experiments to come. We found that adding 0.2 mg/ml bovine serum albumin (BSA) was sufficient to prevent nucleosome degradation up to several hundred mM NaCl [46] but failed to do so at higher ionic strength. Better passivation was achieved by small amounts of non-ionic surfactant added into solution; for most of our more recent work, we employed a combination of 0.01% Nonidet P-40 and silanization of the bottom glass surface of a microplate [47].

Adding unlabeled nucleosomes into solution proved to be an elegant way to maintain single nucleosome detection sensitivity at ensemble-like conditions, as only the labeled subset of nucleosomes (typically 20–50 pM) is detected. First, this helps to better compare results from single molecule experiments with ensemble measurements, as overall nucleosome concentrations can be adjusted to the same value (a concept we termed “quasi-bulk FRET”, discussed in a more recent paper, which dealt with ways to bridge the experimental gap between classical bulk and single molecule experiments [47]**.** Second, by progressively lowering the concentration of unlabeled complexes, nucleosomes can be destabilized in a controlled manner, shedding light on the architectural changes nucleosomes undergo upon disassembly, a process that moved into the focus of Jörg Langowski’s nucleosome-related research in the last decade. Nucleosome disassembly is essential to overcome the steric barrier that nucleosomes pose to DNA-processing enzymes during transcription, replication and DNA repair.

In vivo studies have shown that the speed of polymerase transcribing through the nucleosome is similar to that measured on free DNA. Therefore, an efficient mechanism has to ensure rapid disassembly of nucleosomes in front and reassembly of them behind the elongating polymerase to maintain chromatin integrity [46].

In vitro experiments often induce disassembly by raising the ionic strength in solution – a method that has become well-established to analyze nucleosome stability. It is generally believed that intermediate species observed at higher ionic strength are similar to those which are transiently populated in the cellular context [48]. By the end of the last decade, little was known about the existence and dynamic properties of intermediate structures that are populated during nucleosome disassembly and we began a systematic research effort into nucleosome disassembly, leading to more than 7 papers on various aspects of this process – from establishing an initial model for the disassembly pathway to its detailed kinetic analysis to the role of posttranslational modifications and protein mutations on nucleosome opening. Since the study of the dynamics of nucleosomes, also through FRET methods, is one of the major future directions taken by the Langowski’s lab, I will review this work in more detail in the following.

#### Assembly and disassembly nucleosomes dynamics

As a first step towards a detailed analysis of disassembly, we compared nucleosomes with end-labeled and internally labeled DNA using two different nucleosome positioning sequences, the naturally occurring 5S rDNA sequence and the artificial 601 sequence, which comprises one of the strongest positioning sequences to date. It was evident that disassembly commences by increased dynamics of the linker DNA ends. At physiological ionic strength both internally labeled nucleosomes show very similar conformations, while sequence-specific differences were observed in the entry/exit region of the DNA. The strongly positioning 601 sequence shows a much narrower distribution than the 5S rDNA, indicating that the latter sequence has more conformational freedom and frequently assumes a more compact structure, in agreement with reduced DNA unwrapping observed by North et al. [49]. At higher salt, nucleosomes progressively disassemble from the ends. FRET between the DNA ends is lost at lower ionic strength than FRET between internal sites on the DNA (Fig. 4).

This initial work was followed by a thorough spFRET study of nucleosome disassembly in collaboration with the lab of Prof. Claus Seidel in Düsseldorf [28]. Nucleosomes were reconstituted on a 170 bp DNA fragment containing the 601 positioning sequence, with both fluorophores placed in the central region of the nucleosome. For the first time, multiparameter fluorescence detection (MFD) [50] was used on individual nucleosomes and revealed the coexistence of three states with different FRET; intact, fully compacted nucleosomes, a partially opened intermediate species and fully open DNA. This work successfully established a model for stepwise disassembly through loss of H2A/H2B dimers, which was also proposed in related or similar studies by other groups [50–56], yet the exact sequence and mechanism of disassembly still remained unknown at the time.

An important step that further enhanced our understanding of nucleosome disassembly was the discovery of a previously unknown intermediate structure, which today is often referred to as the “butterfly-state”. During our initial work we realized that labeling various sites of nucleosomal DNA alone does not suffice to fully understand the complex dynamics of nucleosome disassembly. More sophisticated experiments and labeling schemes were needed to systematically vary the position of the reporter dyes on DNA and protein. By monitoring distance changes between various regions of the nucleosome a more global picture of nucleosome disassembly could thus be obtained, not unlike the concept of modern navigation through GPS.

In her PhD thesis, Vera Boehm expanded the set of labeled nucleosome constructs to samples labeled on DNA and histone proteins H2B and H4. From the variation of FRET efficiency with NaCl concentration she could propose a new intermediate state where all histones were still associated with DNA but the nucleosome core was opened up at the dimer:tetramer interface. This structure, reminiscent of a butterfly with spread wings, appears to be a general hallmark of disassembly; in a follow-up study we could show that dissociation through this intermediate state was observed for different DNA sequences and histone protein origins [56]. Additional support for nucleosome conformations with partially disrupted dimer-tetramer contacts was later provided by other groups using time resolved small-angle X-ray scattering (SAXS) data [57] or similar spFRET studies [58].

In vivo, DNA topology also impacts nucleosome disassembly and genetic activity. Cellular chromatin can experience transient torsional stress, in particular during transcription, where positive superhelical torsion is generated in front of and negative supercoiling behind the elongating polymerase [57]. It has been observed that during transcription nucleosomes are displaced downstream and re-loaded upstream from the polymerase [59], often accompanied by partial or complete histone loss [60, 61]. Until recently, it was not clear whether this was directed by the topological change in DNA or by torsion-related effects on associated proteins. While earlier ultracentrifugation studies suggested that positive or negative supercoiling had no effect on DNA compaction by nucleosome formation [62], another PhD student of Jörg’s, Tabea Elbel, conclusively proved that positive superhelical torsion can indeed destabilize nucleosomes in accordance with another study [63]. Elbel and Langowski studied the effect of superhelical density on the dissociation of nucleosomes assembled on plasmid DNA. Combining scanning force microscopy and FCS data they found direct proof that positive torsion promotes H2A-H2B heterodimer loss [64]. Sheinin et al. arrived at the same conclusion through indirect observation of dimer eviction in mechanic force experiments [65].

Eventually, the kinetics of nucleosome disassembly and the effect of posttranslational modifications came into focus of Jörg’s research. We initially characterized the kinetics of salt-induced disassembly on a minute time scale [28], and intermediate structures over similar time scales have also been reported in a recent spFRET study by Hazan et al. [66] from Eyal Nir’s group, a work which was externally co-supervised by Jörg Langowski. Unraveling dynamic processes on much smaller time scales, however, requires the ability to monitor changes in FRET efficiency while the nucleosome diffuses through the laser focus. Over the last years the group of Claus Seidel, a long-time collaborator of us developed a set of methods and theoretical approaches to extract this dynamic information from diffusing molecules in solution. Extending our successful collaboration we applied their latest advances in single molecule analysis to investigate spontaneous structural fluctuations within nucleosomes. Multiparameter single molecule fluorescence [50, 67] analysis of nucleosomes labeled at multiple positions revealed a rapid interconversion between partially disrupted conformations, where the nucleosome fluctuates between open and closed octasome (all proteins bound to DNA) and hexasome (missing one H2A-H2B dimer) structures. In combination with species-selective FCS we were able to develop a full kinetic scheme of nucleosome disassembly with at least 7 separate species that intercovert on time scales ranging from microseconds to minutes (Gansen et al., in revision) and to provide hypothetical geometries of the intermediate states, where data was most consistent with an opening of the dimer:tetramer interface by 20–25°. These results mark the most detailed kinetic scheme reported for nucleosomal processes so far.

#### Regulation of nucleosome dynamics through histone tails and posttranslational modifications

Canonical nucleosomes with unmodified DNA and proteins provide a pure model system to study the dynamic landscape of nucleosomes. In the biological context, however, nucleosomes carry a multitude of chemical modifications which can affect their dynamic properties and have been recognized in the last 15–20 years as a key element in gene regulation [68]. They can affect chromatin morphology by altering electrostatic DNA-protein interactions, nucleosome stability and nucleosome-nucleosome interactions and form binding epitopes for chromatin-associated factors that can further modulate genetic activity.

Many of these modifications target the unstructured histone tails, which comprise about 30% of the total histone mass. These disordered regions feature a large number of basic residues, such as lysines or arginines, which can be acetylated or methylated. Most recent work in the Langowski lab focused on the effect of histone tail modification on nucleosome architecture.

The exact interplay of the long N-terminal tails of H3 and H4 is still a matter of debate. Crosstalk of posttranslational modifications (PTMs) between both tails has been observed [69], where the presence of PTMs on one tail can affect the deposition of modifications on the other tail. Earlier bulk work has shown that partial removal of the H3 and H4 tails increased site exposure to nuclear proteins, but that the combined removal of different tails does not necessarily have a synergistic effect [70]. A more recent study suggested that simultaneous removal of H3 and H4 tails increases DNA unwrapping compared to clipping either tail individually [71]. Earlier bulk experiments on chemically acetylated histone tails from our lab, however, challenged the idea of a purely synergistic interplay between H3 and H4. The data obtained by Katalin Tóth on end-labeled nucleosomes suggested that H3-acetylation led to an increase of DNA end-to-end distance but H4-acetylation had the opposite effect [72]. In how far these differences reflect an overall change in stability or an increase in linker end-to-end dynamics remained unclear. To address this question in more detail, we recently performed a detailed analysis of the role of histone acetylation on DNA unwrapping and disassembly. Using a combination of bulk FRET and spFRET, we further established the antagonistic role of H3 and H4 acetylation in nucleosome stability [73]. Acetylation of histone H3 opened up the nucleosome ends and promoted disassembly, but its destabilizing role was counteracted by H4-acetylation in nucleosomes with both histones acetylated. Despite significant differences between the various acetylated constructs, however, the overall effect of histone tails on stability is more subtle than for example the impact of DNA sequence, but can provide regulatory fine tuning of nucleosome stability. The special role of histone H4 that emerged from these studies clearly warrants further investigation into its role in nucleosome architecture.

It is not too surprising that modifications on the histone tails have fairly subtle impact on nucleosome stability. Stronger effects on nucleosome stability are expected for mutations within the histone core domains, as these do more directly affect DNA-protein and protein-protein interactions that hold the nucleosomes together.

Recent work from the Poirier lab systematically investigated the role of specific residues in the histone-fold region of the nucleosome by chemically introducing point mutation at defined positions in the histone core [74]. The authors could show that the change of a single amino acid within the nucleosome is already sufficient to significantly alter its dynamic properties. A combination of magnetic force spectroscopy and FRET revealed that PTMs near the dyad axis (H3K115ac and H3K122ac) selectively affected nucleosome disassembly without impacting unwrapping at the ends, while the opposite was observed for modifications in the entry/exit region (H3K56ac, H4K77ac and H4K79ac). These studies imply that histone-DNA interactions are decoupled to selectively modulate different dynamic processes within the nucleosome.

From these studies it has become clear that with so many amino acids that are potentially involved in nucleosome stability, a purely experimental approach to study their role is tedious. In silico approaches, which could screen many of these residues for potential impact, are of great interest. It has always been Jörg’s vision to combine modeling approaches with experimental data to learn more about nucleosome and chromatin dynamics. For many years, however, this has been impossible to achieve, mostly because of the lack of appropriate details in the simulations. With increasing computational capacity, it is now possible to perform all-atom simulations of nucleosomes over an extended time scale and to directly link these findings with structural data from single molecule experiments. When combined with state-of-the-art single molecule experiments they can thus provide more insight into the role of individual PTMs on nucleosome architecture.

Here, the interplay between the electrostatic environment near the H2A/H2B interface and nucleosome integrity was linked to histone tail dynamics and their modifications. Modeling studies correlated histone tail removal with increased DNA unwrapping [31] and structural transitions in the nucleosome core [30]. Similar results were recently obtained in Jörg’s lab, where a conservative mutation of two arginine residues in histone H2A located at the interface between dimer and tetramer dramatically reduced nucleosome stability and promoted formation of intermediate structures during disassembly [29].

In the last 20 years it has been become evident that nucleosomes – once thought to be a purely static component in DNA compaction – represent highly dynamic entities that have profound impact on gene regulation. From early biochemical and crystallographic experiments to recent high-resolution FRET data obtained by Jörg Langowski’s group and many other groups worldwide we gained enormous insight in the overall structural properties of nucleosomes. With the advent of modern microscopic techniques and advanced data analysis it has became possible to follow nucleosome dynamics on sub/ms time scales and to identify a multitude of interconverting sub-states that have previously been anticipated only by theoretical considerations.

## Conclusions: Not an end

Jörg Langowski had no interest in conforming to the mainstream, and had strong opinions on science and its communication. Overall, his legacy has been to propel a way of viewing biological function that was not popular when he started pushing it: an approach that goes beyond sequences and gels, viewing living systems as dynamic and in three dimensions. This physical view of biology took a long time to take root, in part because the average biologist does not have the capacity that Jörg had to marry physical science with biology. It is now, finally, becoming established.

What is the future for chromatin and chromosome structure? Young scientists, new techniques, more powerful computational analyses of deep databases and more sophisticated molecular modeling. From all of these, we can expect great advances in understanding the plethora of chromatin polymeric states. Jörg would have enjoyed the exciting future. We would have enjoyed discussing it with him.

Jörg’s contributions to science are also the hundreds of students who worked under his supervision or attended his talks. He was never tired to passionately introduce new students into his scientific interests and his door and ears stayed open for everybody’s questions. He gave a lot of freedom to the students, even more, he demanded independent thinking and working. The interdisciplinarity of research topics of the group required that physicists, medical doctors, mathematicians, chemists and biologists had to learn each other’s language and could thus debate results.

**Fig. 12:**
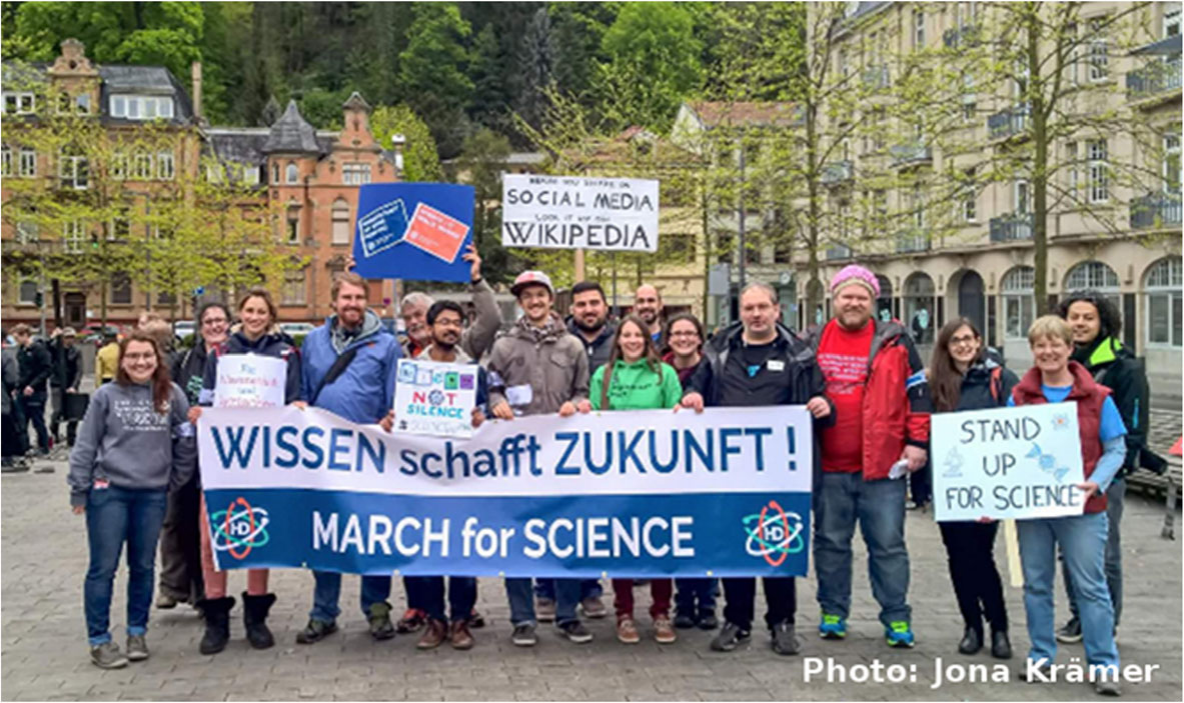
Jorg at the macrh for science. http://www.chemistryviews.org/details/ezine/10533299/The_March_for_Science_Continues.html

Jörg strongly believed in open access journals and was an editor for PloS One and BMC Biophysics. The freedom of research was a very important issue for Jörg. In spring 2017, he went on the barricades for it (Fig. 12), and wholeheartedly helped to organize the March for Science and a follow up discussion. He fought for the open access of all results, believed that only this can promote science even on the cost of personal impact factors. Competition-free and fruitful conferences, that he organized on this basis and co-operations in which he always gave all of his knowledge show what can be achieved with an open mind like his.

Scientific adventures: Jörg believed and proclaimed that solely adventures can lead to revelations while planning requires knowing the result in advance. Besides curiosity, adventures need a lot of courage and self-confidence, which he was never short of. At the molecular level, adventures parallel the random motion, diffusion, which were the central topic of all his research.

## Abbreviations

AFM: Atomic Force Microscope
AML: Acute Myeloid Leukemia
AUC: Analytical ultracentrifuge
BD: Brownian Dynamics
BSA: Bovine Serum Albumin
ELCS: Nuclear Envelope-Limited Chromatin Sheets
FCCS: Fluorescence Cross-correlation Spectroscopy
FCS: Fluorescence Correlation Spectroscopy
FFM: Fluorescence Fluctuation Microscope
GFP: Green Fluorescent Protein
LAD: Lamina Associated Domain
MD: Molecular Dynamics
PCS: Photon Correlation Spectroscopy
PTMs: Post-Translational Modifications
RA: Retinoic Acid
SAXS: Small-Angle X-Ray Scattering
SEC: Sub-Elastic Chain
spFRET: Single Protein Foerster Resonance Energy Transfer
SPIM: Single Plane Illumination Microscopy
TAD: Contact Domains, Compact Domain
WLC: Worm-Like Chain
